# The Spanish Version of the Childhood Anxiety Sensitivity Index: Factorial Dimensions and Invariance across Gender in a Sample of Adolescents

**DOI:** 10.3390/ijerph20043045

**Published:** 2023-02-09

**Authors:** Paloma Chorot, Rosa M. Valiente, Bonifacio Sandín

**Affiliations:** Facultad de Psicología, Universidad Nacional de Educación a Distancia (UNED), 28040 Madrid, Spain

**Keywords:** Childhood Anxiety Sensitivity Index (CASI), adolescents, anxiety sensitivity, factor analysis, gender

## Abstract

Anxiety sensitivity is a transdiagnostic construct that has been associated with the etiology of emotional disorders, especially panic and other anxiety disorders. Although it is well known that, for the adult population, the factor structure of anxiety sensitivity includes three separate facets (physical, cognitive, and social concerns), the facets of anxiety sensitivity for adolescents have not yet been established. The main goal of the present study was to examine the factor structure of the Spanish version of the Childhood Anxiety Sensitivity Index (CASI). A large sample of nonclinical adolescents completed the Spanish version of the CASI in school settings (*N* = 1655; aged 11–17 years; 800 boys and 855 girls). Exploratory and confirmatory factor analyses of the full scale (CASI-18) indicate that a three first-order factor solution represents appropriately the three anxiety sensitivity facets previously defined for the adult population. The 3-factor structure had a better fit and was more parsimonious than a 4-factor solution. Results also indicate that the 3-factor structure remains invariant across genders. Girls scored significantly higher than boys on the total scale and on all three dimensions of anxiety sensitivity. In addition, the present study provides information concerning normative data for the scale. The CASI holds promise as a useful tool for assessing general and specific facets of anxiety sensitivity. It could be helpful for the assessment of this construct in clinical and preventive settings. The limitations of the study and suggestions for further research are outlined.

## 1. Introduction

Anxiety sensitivity was first defined by Reiss and McNally [1] as an individual difference variable. It is described as the fear of anxiety symptoms that arise from beliefs that the experience of fear/anxiety causes illness, embarrassment, or additional anxiety. Thus, anxiety sensitivity arises from beliefs that anxiety symptoms have harmful consequences. For example, people with high anxiety sensitivity may believe that rapid heartbeats signify an impending heart attack, whereas people with low anxiety sensitivity will regard such sensations as merely unpleasant. Anxiety sensitivity has been suggested as a risk factor for the development of panic disorder and other emotional disorders [2,3] and is currently considered a transdiagnostic construct [4,5,6]. Anxiety sensitivity is conceptually and empirically distinct from general trait anxiety; anxiety sensitivity denotes the tendency to respond fearfully to anxiety symptoms, whereas trait anxiety denotes the tendency to respond fearfully to stressors in general [7,8].

Anxiety sensitivity has been largely assessed in adults by means of the Anxiety Sensitivity Index (ASI), a measure originally developed by Reiss et al. [9]. Factorial studies based on this scale have shown that anxiety sensitivity is a multidimensional construct consisting of three lower-order factors loading on a high-order factor [2,10,11,12]. The three lower-order factors were defined as “physical concerns”, “mental incapacitation concerns”, and “social concerns” [11]. The Anxiety Sensitivity Index-3 (ASI-3) [13] is a new 18-item self-report multidimensional measure based on the original ASI developed by Reiss et al. [9]; it was designed to make a balanced assessment of the three basic dimensions of anxiety sensitivity, i.e., physical concerns, cognitive concerns, and social concerns. This measure has been shown to have a stable hierarchical multidimensional structure (i.e., three lower-order factors loading into a higher-order factor) and sound psychometric properties, with the three primary factors showing theoretically consistent patterns of association with anxiety symptoms and diagnoses [14].

The Childhood Anxiety Sensitivity Index (CASI), developed by Silverman et al. [15], is a modified version of the ASI [9] to assess anxiety sensitivity in children and adolescents. It consists of 18 items, 16 of which are similar to items in the adult ASI, with 2 items added (the first 16 items show identical correspondence to the first 16 items on the ASI). The CASI is now a very international measure, having been translated into major languages. Likewise, several types of studies focusing on the CASI have shown that it has sound psychometric properties, including reliability, discriminant and predictive validity, and clinical utility [15,16,17,18,19,20]. However, a main problem concerns the factor structure of the scale [20].

Although it is assumed that the CASI also has a hierarchical structure consisting of multiple lower-order factors (primary factors) loading on a higher order factor [16,17,18,19], the main current problem is that it is not clear what the primary factors are. In addition, there is also no agreement regarding the variables that configure each factor. The recent systematic review of the factor structure of the CASI conducted by Francis et al. [20] suggests that, based on the 18 factor analytic studies reviewed (12 of which used the full scale), the nature of the structure of this measure (primary factors) is controversial. According to these authors, the review yielded many discrepant findings, with little cohesiveness or consistency among the results of different studies. From these studies, the most identified factor structure of the CASI was a 3-factor model that, in line with the structure of the ASI and ASI-3, was comprised of the factors of physical concerns, mental incapacitation concerns, and social concerns. This three-factor structure is, in general, consistent with the three-factor model first found empirically by Silverman et al. [16] with mixed samples of children and adolescents (i.e., physical concerns: items 3, 6, 9, 10, 11, 14, 16, and 18; mental incapacitation concerns: items 2, 12, 13, and 15; and concerns about publicly observable symptoms: items 1, 4, 5, 7, 8, and 17). This model was supported by nine studies (two of which provide support for both three- and four-factor structures), with some item configural variations across studies.

In a subsequent study based on a 13-item version of the CASI, Silverman et al. [17] found the best fit for a 13-item version of the CASI representing a hierarchical model with a single second-order factor and four first-order factors, which they labeled as disease concerns (items 3, 6, 9, and 11), unsteady concerns (items 4, 8, and 10), mental illness concerns (items 2, 12, and 15), and social concerns (items 1, 5, and 17). This 4-factor model was the next most frequently reported model in the review of Francis et al. [20], but it was generally associated with the 13-item version of the scale (i.e., items 7, 13, 14, 16, and 18 were not included in the scale). The results found by the remaining studies reviewed by Francis et al. [20] were heterogeneous, suggesting a factor structure composed of one [21], two [22,23], or five [24] first-order factors.

Apart from the inconsistency of the studies regarding the number of retained factors and considering that some authors have shortened the scale, there is no agreement on which items are unsuitable, if any. As noted above, although most studies used the full 18-item scale, other versions including only 13 (four studies), 16 (one study), and 9 (one study) items have also been used. Another important problem observed in the literature is that most of the studies used mixed samples of children and adolescents; only 5 of the 18 studies reviewed by Francis et al. [20] were based on samples of adolescents. This could generate serious doubts regarding the interpretation of results because some items may have different meanings for children and adolescents.

The preliminary data from the Spanish version of the CASI applied to children suggest that the scale has a structure of three primary correlated factors [25]. This 3-factor model is similar to that described by Silverman et al. [16] using a mixed sample of children and adolescents; there were only discrepancies in the loading of three items. This study also suggests a hierarchical structure based on these three primary factors and one higher-order factor. Although both oblique models seemed to fit the data well, in line with the results found by Muris et al. [26], the non-hierarchical model had a slightly better fit [25]. To our knowledge, data on the factorial structure of the Spanish version of the CASI based on samples of adolescents have not yet been published.

Published data based on adolescent samples are inconclusive. Only 5 out of the 18 factor studies included in the systematic review of Francis et al. [20] specifically targeted the factor structure of the CASI in adolescents [18,20,21,26,27,28], and only 3 [18,27,28] assessed the full 18-item version of the scale. The remaining 2 studies focused on shortened versions, including a 16-item version equivalent to the ASI for adults [26] and a 9-item version comprising the physical factor [21]. Even though Adornetto et al. [27] stated that the 4-factor structure based on the 13-item version of the CASI (Silverman’s model) was the best factorial solution, they did not really find significant differences, neither between the 3- and 4-factor structures nor between the hierarchical and non-hierarchical correlated models. Likewise, Brown et al. [28], using the 18-item version of the scale, found that 3- and 4-factor models (both hierarchical and non-hierarchical) revealed a largely comparable fit to the data. However, as these authors point out, a 3-factor solution representing physical, social, and mental concerns was favored because the 4-factor model represented an over-extraction of factors. The 3-factor solution has a comparable fit but greater interpretability and parsimony. This model clearly delineates the three distinct facets of anxiety sensitivity defined from adult samples and describes the data with the fewest number of factors. Finally, the study of Essau et al. [18], based on the complete version of the CASI (18 items), highlighted that, through exploratory and confirmatory factor analyses, a 3-factor solution (conceptually equivalent to Silverman’s model) [16] best explained the data. Each of the three lower-order factors (physical concerns, mental incapacitation concerns, and social concerns) loaded onto a higher-order factor. An important conclusion of this work was that the 3-factor model was preferable to the 4-factor model because it is more parsimonious than the latter and has a better fit.

As can be seen, the reported data on the structure of the CASI in adolescents are controversial. Among the main problems of past research, the following should be highlighted: (a) most studies have used mixed samples of children and adolescents (very few studies have been based on separate samples of adolescents); (b) use of multiple versions of the CASI regarding the number of items (versions of 9, 16, 13, and 18 items have been used); and (c) inconsistency in the number of isolated primary factors (structures of one, two, three, four, and five lower order factors have been proposed).

Therefore, a first issue arises as to the factor structure of the CASI, and more specifically, to examine the primary factors of scale. Consistent with the theoretical and empirical rationale for anxiety sensitivity [2,9], including childhood anxiety sensitivity [18,20,28], we expected that a 3-factor solution would better represent the primary dimensions of the CASI. Because the empirical evidence on the factorial structure is not consistent, it is difficult to make a firm prediction, although the few studies that used samples of adolescents seem to suggest the relevance of a three-factor structure.

Another potential problem related to research on the factorial structure of the CASI concerns the characteristics of the sample utilized. With few exceptions, mixed samples of children and adolescents have generally been used. Some of the inconsistencies found in previous studies could be associated with possible differences due to age; for example, some items on the scale may not be understandable to younger children. Furthermore, many studies used too small samples, which could influence the consistency and statistical power of the data. Therefore, a second goal of the present study was to test the structure of the CASI using a large sample of adolescents.

A third purpose of this study was to test the invariance of the factor structure of the scale across gender. There is evidence that girls score higher than boys on several facets of the CASI, and examination of the factor structure as a function of gender would ensure that the previously observed gender differences in childhood anxiety sensitivity are due to actual differences in the relative focus of concerns between girls and boys and not due to gender differences in the CASI factor structure [29]. Although some authors [16,30] found the factor structure to be relatively invariant across gender, others [27] have not documented such invariance.

Finally, a fourth objective concerned the validation of the factorial structure of the Spanish CASI in a sample of adolescents. In our previous work on the factor structure of this scale [25], we used a sample of children ranging in age from 9 to 11 years. Given the high interest on this scale in the Spanish-speaking population, its validation with adolescents was needed.

## 2. Materials and Method

### 2.1. Participants and Procedure

The large sample consisted of 1655 nonclinical adolescents (800 boys and 855 girls) aged 11–17 (mean age = 14 years, *SD* = 1.9). The children were recruited from secondary schools in the Community of Madrid (Spain) and attended regular classes. Concerning parental education, 19% had not finished high school, 40% had a high school certificate, and 38% had a college degree. Three percent of participants did not provide information. Most of the sample was Caucasian (89%). The remaining were Hispano-American (10.1%), Asian (0.60%), and Black (0.30%). Schools were enrolled with the consent of the headmaster and school board. To maximize consent and participation, the study was offered to the adolescent and parents in the context of a course of education on emotional health. Informed consent forms from parents were obtained by the school’s staff. Adolescents and teachers gave their verbal consent to participate on the day of the test. All participants completed the questionnaires at school during regular class hours.

### 2.2. Measures

The Childhood Anxiety Sensitivity Index (CASI) [15]; Spanish version by Sandín [31] (see Table A2): self-report questionnaire that assesses the fear of anxiety symptoms in children and adolescents. For each of its 18 items, adolescents rated on a 3-point Likert scale, ranging from 1 (“none”) to 3 (“a lot”), the extent to which they believe the experience of anxiety will result in negative consequences for them. The CASI yields a total score by summing the ratings across all 18 items; higher scores denote higher levels of anxiety sensitivity. Previous studies with the Spanish version of the CASI have shown adequate psychometric properties for nonclinical samples. For example, this version has demonstrated that the CASI is best viewed as a multidimensional scale of three correlated first-order factors pertaining, respectively, to physical, mental, and social concerns [25]. The reported internal consistency estimates (α coefficient) range from 0.80 to 0.89 [25,32,33]. Evidence has also been provided on its convergent, discriminant, and predictive validity, having demonstrated its uniqueness with respect to other related measures, such as fear frequency, trait anxiety, and negative affect [32,33,34]. 

### 2.3. Statistical Analysis

The data were analyzed using EQS 6.3 and SPSS 25 statistical programs. Initially, in order to determine the number and nature of the lower-order factors, we conducted exploratory factor analysis (EFA) based on the polychoric correlations between the variables. The method of extraction of least squares (LS) and oblique rotation (GEomin) were used. The number of factors to retain was determined through parallel analysis (based on the mean and the percentage [95%] of the eigenvalues on 1000 sets of simulated random normal) and the scree test (based on a plot of the eigenvalues). Following Taylor et al.’s suggestions [35], we also used Thurstone’s criteria: (1) a minimum number of items with salient loadings (>0.30) on more than one factor (i.e., a minimum number of “complex items”); (2) a minimum number of items that do not have salient loadings on any factor (i.e., a minimum number of “hyperplane items”); and (3) each factor is well-defined (i.e., it has three or more salient loadings per factor). We also used the criterion of theoretical interpretability of the resulting factor structures [36].

In the second step, we conducted confirmatory factor analyses (CFA) using the robust method of maximum likelihood estimation (MLR) based on polychoric correlations. The starting models to be tested were defined according to the results of the EFA as well as the 1-factor structure. To assess the adequacy of each model, we applied the following fit indices: the Satorra–Bentler scaled chi-square (S-Bχ^2^)/df (values ≤ 5 of the S-Bχ^2^/df ratio indicate adequate fit; lower values indicate better fit), the goodness-of-fit index (GFI), comparative fit index (CFI), the standardized root mean square residual (SRMR; absolute index), the root mean square error of approximation (RMSEA; parsimony-corrected index), and Akaike’s information criteria (AIC). The following cut-off criteria are commonly considered to represent a well-fitting model: GFI and CFI ≥ 0.90, SRMR ≤ 0.08, and RMSEA ≤ 0.05. The AIC is suitable for comparing competing models; the model with the lowest AIC is potentially considered the best model [37].

The reliability (internal consistency) of the CASI was examined by means of structural equation modeling procedures (omega and rho). When used with a multifactor model, the rho coefficient provides a good estimate of internal consistency and is a recommended coefficient [38]. When used with a single factor model, rho is the same as McDonald’s omega coefficient. 

## 3. Results

### 3.1. Exploratory Factor Analysis (EFA)

Because there is still controversy about the exact number of lower-order factors in the CASI, it was necessary to previously perform an exploratory factor analysis (EFA) of the data. Using the selected extraction criteria (parallel analysis, scree test, and interpretability of the factor structure), we obtained a 3-factor solution as the best choice. Accordingly, we extracted a factor structure of three first-order correlated factors, as summarized in Table 1. As can be seen, the first factor (items 3, 4, 6, 8, 9, 10, 11, 14, and 18) includes items that pertain to physical concerns; the second factor (items 2, 12, 15, and 16) is defined by CASI items about cognitive (mental) concerns; and the third factor (items 1, 5, 7, 13, and 17) represents the dimension of social concerns. This factor structure seems strong since there are no cross-loadings and each factor is well defined. Likewise, such a structure is theoretically consistent. Perhaps a possible exception concerns to Item 16 (“It scares me when I feel nervous”), which has a general (decontextualized) formulation and so it could be assigned to any of the three factorial dimensions. Accordingly, for further studies, we suggest rewording this item as follows: “When I feel nervous, it scares me that I can’t keep my mind on what I’m doing”.

Correlations among the three factors (polychoric correlations) and among the corresponding CASI subscales (observed measures) were calculated (see Table 2). We found moderate correlations between the factors as well as between the subscales, and large correlations with the CASI total score. The reliability coefficients denote good levels of internal consistency in the total scales and subscales, except for the social concerns subscale (Table 2). 

### 3.2. Confirmatory Factor Analysis (CFA)

Through CFA, we examined the correlated 3-factor model empirically established as the best model in the previous EFA. We also examined three alternative models, i.e., a single factor model, a 3-factor orthogonal model, and a hierarchical model (consisting of three lower-order factors loading on a higher-order factor). The one-factor model was examined because there is some evidence of strong unidimensionality for the CASI [16,17,18,19,20]. In addition, the four models suggested by Silverman et al. [16,17] were also tested. We examined Silverman’s models because they have been validated in several studies, especially the 3-factor correlated/hierarchical models [20]. Using the modification indices of CFA, the error covariances of two pairs of overlapping items (E6, E9 and E14, E18) were freely estimated. Table 3 shows the fit indices corresponding to these tested models. 

Models 3 and 4, i.e., the model of 3 correlated factors and the hierarchical model (3 lower-order factors loading on a higher-order factor), showed similar and good fit to the data (see Table 3). Although both models show largely comparable fits, the correlated 3-factor solution was chosen on the grounds of comparable fit and greater parsimony. Thus, the model of three correlated factors (Model 3) was used to create subscales reflecting physical, cognitive, and social concerns and for further analyses. The factor loadings (fully standardized coefficients) corresponding to this model are shown in Table 4. Most factor loadings are ≥0.58, although Item 17 (“I don’t like to let my feelings show”) did not reach the value of 0.30. Based on fit indices and factor loadings, we can conclude that, in general, the model has an excellent fit and seems to adequately represent the factorial structure of the CASI.

The goodness-of-fit indices for the tested Silverman models are presented in Table 3. As can be seen, the models for the CASI-13 showed a better fit in general than the CASI-18 models. However, the fit indices for these 2 models (i.e., models for CASI-13) were only slightly better than for the correlated 3-factor model of the CASI-18 (Model 5), and largely similar to Model 3 (i.e., the model of 3 correlated factors that was defined in the present study).

### 3.3. Invariance across Gender

We tested the equivalence of the correlated 3-factor model of the CASI across the samples of boys (*n* = 800) and girls (*n* = 855). It was examined by confirmatory multigroup factor analysis. To this purpose, we examined if the participants of the boys’ and girls’ samples responded to the items of the CASI in a way that produced an equal number of factors and factor-loading pattern (invariance of the configural model), equal factor loadings (invariance of the measurement invariance), and equal factor variances and covariances (invariance of the structural model) [37]. In testing for gender multigroup invariance, the fit of the invariance models yields a set of statistics for overall model fit. It is argued that invariance holds if goodness-of-fit related to the model is deemed adequate [37]. Results of this analysis show that the correlated 3-factor model of the CASI represents a good fit to the data across genders (see Table 5). As can be seen, both measurement and structural multigroup constrained models still represent a good fit to the data despite the imposition of these equality constraints, with a negligible difference in fit from that of the configural model.

### 3.4. Descriptive Statistics

Descriptive statistics (means and *SDs*) for the CASI and the three subscales are shown in Table 6. Statistically significant differences were found in the means between boys and girls. Normative values (percentile cut points) for scores on the CASI subscales and the CASI total are indicated in Table A1.

## 4. Discussion and Conclusions

The general objective of the present study consisted of providing evidence on the construct validation (factor structure) of the Spanish version of the CASI in a large sample of nonclinical adolescents. More specific objectives were: (1) to identify the lower-order factors of the scale and confirm the best primary structure by means of confirmatory factor analysis; (2) to examine the factorial invariance of the CASI across gender; and (3) to provide normative and cut-off (percentile) scores.

In relation to the first objective, the data based on exploratory and confirmatory factor analysis suggest that a three-correlated factors solution is best explained. This 3-factor structure (Model 3) is theoretically coherent and represents the three anxiety sensitivity facets that consistently have been reported from adult samples [2,11,13,14,39], i.e., the dimensions of physical, cognitive, and social concerns. Although several authors have suggested a factor structure of four correlated factors [16,17,19,27], the results are in line with the conclusions of Francis et al.’s systematic review [20]. These authors stated that more published factor analytic studies support a three-factor model depicting physical, mental (cognitive), and social anxiety-related concerns. Consistent with suggestions from other studies conducted with adolescent samples [18,28], a three-factor structure, compared to a four-factor solution, represents more clearly the facets of anxiety sensitivity that have been reported in the literature. Likewise, our results suggest that the four-factor solution implied an over-extraction of factors and was less parsimonious.

Although Silverman’s models of the CASI structure have been widely used, it is not clear which version (18 vs. 13 items) and which model (3 vs. 4-factor structure) are recommended to be used. Our Model 3 showed an acceptable match to the corresponding items found by Silverman et al. [16,17] or Essau et al. [18]. Except for Item 13 (“Other kids can tell when I feel shaky”), all other items matched to the first-order factors in Silverman et al. or Essau et al. Only Item 13 belonged to a different factor in the three studies, i.e., social (the present study), cognitive (Silverman et al. [16]), or physical (Essau et al.) concerns. We think that this item is theoretically better related to the social facet of anxiety sensitivity. Thus, the three-factor model (i.e., Model 3) seems to well represent the first-order factors of the full scale (CASI-18). Likewise, our data suggest that the three-factor structure is also appropriate for the short version (CASI-13). However, we have some doubts about the validity of the social subscale of the shortened form [17] due to the suppression of Items 7 and 13; according to the results of the present study, these two items seem to be the ones that best represent the social dimension. 

A second objective consisted of examining the factorial invariance of the CASI at the multigroup level across genders. With respect to this objective, we expected that the structure of the scale would remain invariant, independently of the sample (boys vs. girls). As noted above, results are consistent with this hypothesis and show the structural invariance of the scale regardless of gender. Some studies have yielded findings suggesting that girls and boys may experience anxiety sensitivity differently [16,26,34]. However, research to find out whether anxiety sensitivity has a similar structure across genders is sparse. Some studies based on mixed samples of children and adolescents provided preliminary evidence support that the four-factor [17] and the three-factor [29,30] structures of the CASI is relatively invariant across genders. The only work carried out with a sample of adolescents [27] found that the four-factor structure of the CASI-13 was noninvariant across gender. The present study is the first to examine the invariance of the three-factor structure using the full scale (CASI-18) and a large sample of adolescents. Our data provide evidence to support the idea that anxiety sensitivity is similar in structure across genders in adolescents.

Finally, the present study provides relevant information on the normative data of the Spanish version of the CASI, including means, standard deviations, and percentiles. We found significant differences between boys and girls in the CASI scores as well as in the three subscales, with girls always having higher scores. These results are consistent with data reported in the literature, which show that girls tend to score significantly higher than boys in anxiety sensitivity, both on the total scale and on the different subscales [15,19,26,29,34]. The findings of this study, based on a large sample of adolescents, extend our preliminary data on the validation of the Spanish version of the CASI [25,32,33,34]. Like other versions of the CASI, our Spanish version was characterized by adequate factor structure and good internal consistency. The results may be especially useful for clinical and preventive purposes, especially in Spanish-speaking countries. 

By way of summary, the findings of this study with a large sample of adolescents show that the Spanish version of the CASI has a primary factorial structure of three correlated factors (called physical concerns, cognitive concerns, and social concerns). This structure is consistent with research findings on the structure of anxiety sensitivity in adult samples using the ASI [2,10,11,12] and the ASI-3 [13,14,39]. Although there is still disagreement about the factorial model that best represents the structure of anxiety sensitivity in children and adolescents, an important contribution of the present study was finding that the CASI-18 has a clear structure of 3 correlated factors, this being parsimonious and interpretable (i.e., each item was linked to the theoretically associated factor). This is also the first study to demonstrate that such a factor structure is invariant across gender in adolescents. An examination of the factor structure as a function of gender could ensure that the observed gender differences in adolescent anxiety sensitivity are due to actual differences in the relative focus of concerns between boys and girls and not due to gender differences in the structure of the CASI [29]. Since the factor structure of the CASI was the same for boys and girls, we may assure that gender differences in the CASI subscales are a question of degree. Another strength of the study was to provide relevant information on the validation of the Spanish version of the CASI, with implications for promoting its use in clinical and nonclinical settings.

Although this study has several strengths, a number of limitations are worth noting. First, since the sample was recruited from several schools in the Community of Madrid, it is a non-randomly selected convenience sample. Therefore, it would be desirable to replicate these results with randomly selected samples belonging to other regions of Spain and other Spanish-speaking countries. Second, given the cross-sectional nature of the data, longitudinal studies are needed to determine the nature and course of the CASI structure. Third, the sample was drawn from a nonclinical population; thus, the generalization to clinic populations should be made with caution. Future studies should examine (a) the convergent, discriminant and predictive validity of these three facets of anxiety sensitivity and (b) the clinical meaning of these domains of the CASI, for example, exploring their associations with emotional symptoms and disorders.

## Figures and Tables

**Table 1:** Exploratory factor analysis (factor loadings) of the CASI after GEomin rotation (*N* = 1655).

CASI Item	Factor 1: Physical	Factor 2: Cognitive	Factor 3: Social	*h* ^2^
1. I do not want other people to know when I feel afraid.	0.03	0.13	**0.52**	0.27
2. When I cannot keep my mind on my schoolwork, I worry that I might be going crazy.	0.01	**0.90**	0.02	0.79
3. It scares me when I feel shaky.	**0.47**	0.05	0.21	0.40
4. It scares me when I feel like I am going to faint.	**0.53**	0.03	0.16	0.42
5. It is important for me to stay in control of my feelings.	0.08	0.08	**0.31**	0.16
6. It scares me when my heart beats fast.	**0.63**	0.02	0.03	0.40
7. It embarrasses me when my stomach growls.	0.13	0.15	**0.30**	0.23
8. It scares me when I feel like I am going to throw up.	**0.44**	0.03	0.16	0.32
9. When I notice that my heart is beating fast, I worry that there might be something wrong with me.	**0.78**	0.01	0.11	0.53
10. It scares me when I have trouble getting my breath.	**0.55**	0.01	0.12	0.40
11. When my stomach hurts, I worry that I might be really sick.	**0.57**	0.09	0.05	0.36
12. It scares me when I cannot keep my mind on my schoolwork.	0.01	**0.53**	0.26	0.45
13. Other kids can tell when I feel shaky.	0.28	0.01	**0.36**	0.32
14. Unusual feelings in my body scare me.	**0.68**	0.01	0.07	0.52
15. When I am afraid, I worry that I might be crazy.	0.28	**0.45**	0.01	0.41
16. It scares me when I feel nervous.	0.25	**0.32**	0.23	0.39
17. I do not like to let my feelings show.	0.17	0.01	**0.52**	0.21
18. Funny feelings in my body scare me.	**0.69**	0.04	0.02	0.43
Initial eigenvalues Cumulative proportion of explained variance in data space	6.01 67.63%	1.34 77.84%	1.23 85.05%	

Note. Factor loadings ≥ 0.30 are shown in boldface type. *h*^2^ = communality.

**Table 2:** Correlations between the subscales and between the factors (in parenthesis) of the CASI (*N* = 1655).

CASI Subscales	Physical Concerns	Cognitive Concerns	Social Concerns	CASI Total
Physical Concerns	-----			
Cognitive Concerns	0.52 (0.52)	-----		
Social Concerns	0.44 (0.55)	0.35 (0.29)	-----	
CASI Total	0.91	0.71	0.69	-----
Omega (ω) reliability coefficient	0.86	0.75	0.65	0.88

Note. The rho (ρ) coefficient of reliability was calculated for CASI total.

**Table 3:** Confirmatory factor analysis of the CASI: goodness-of-fit indices for the tested models (*N* = 1655).

*Present Study Models (CASI-18)*	*S*-*Bχ*^2^/*df*	GFI	CFI	SRMR	RMSEA	AIC
Model 1: One factor	4.2	0.853	0.965	0.054	0.045 (0.041–0.049)	294.9
Model 2: Three uncorrelated factors	6.8	0.651	0.936	0.201	0.060 (0.056–0.064)	639.4
**Model 3: Three correlated factors**	**3.1**	**0.903**	**0.978**	**0.048**	**0.036 (0.032–0.040)**	**141.8**
Model 4: Three factors, one higher order factor	3.0	0.910	0.943	0.043	0.058 (0.054–0.061)	139.7
*Silverman’s models*						
Model 5: Three correlated factors, Silverman et al., 1999 (CASI-18)	3.6	0.889	0.972	0.056	0.040 (0.036–0.044)	206.2
Model 6: Four correlated factors, Silverman et al., 1999 (CASI-18)	5.6	0.477	0.951	0.184	0.053 (0.050–0.057)	465.5
Model 7: Three correlated factors, Silverman et al., 2003 (CASI-13 ^a^)	3.2	0.933	0.979	0.048	0.037 (0.031–0.043)	65.0
Model 8: Four correlated factors, Silverman et al., 2003 (CASI-13)	3.1	0.942	0.983	0.045	0.035 (0.028–0.041)	48.7

Note. Corrected indices (robust method). *S*-*Bχ*^2^—Satorra–Bentler scaled *χ*^2^; *df*—degrees of freedom; GFI—goodness-of-fit index; CFI—comparative fit index; SRMR—standardized root mean square residual; RMSEA—root mean square error of ap-proximation; and AIC—Akaike’s information criterion. The robust method was not used to compute GFI and SRMR indices. ^a^: Items 7, 13, 14, 16 and 18 were removed. The selected model is highlighted in boldface type. **Model 3 (items):** physical = 3, 4, 6, 8, 9, 10, 11, 14, 18; cognitive = 2, 12, 15, 16; and social = 1, 5, 7, 13, 17. **Model 5 (items):** physical = 3, 6, 9, 10, 11, 14, 16, 18; mental incapacitation = 2, 12, 13, 15; and publicly observable symptoms = 1, 4, 5, 7, 8, 17. **Model 6 (items):** physical = 3, 6, 9, 11, 14, 16, 18; mental incapacitation = 2, 12, 13, 15; social = 1, 17; and control = 4, 5, 7, 8, 10. **Model 7 (items):** physical = 3, 4, 6, 8, 9, 10, 11; mental illness = 2, 12, 15; and social = 1, 5, 17. **Model 8 (items):** disease = 3, 6, 9, 11; unsteady = 4, 8, 10; mental illness = 2, 12, 15; and social = 1, 5, 17.

**Table 4:** Confirmatory factor analysis of the CASI: fully standardized coefficients (loadings) and squared multiple correlations for the correlated 3-factor model (*N* = 1655).

CASI Factors and Items	Loading	*R* ^2^
*Factor 1. Physical concerns*		
3. It scares me when I feel shaky.	0.64	0.41
4. It scares me when I feel like I am going to faint.	0.68	0.47
6. It scares me when my heart beats fast.	0.58	0.34
8. It scares me when I feel like I am going to throw up.	0.59	0.34
9. When I notice that my heart is beating fast, I worry that there might be something wrong with me.	0.65	0.43
10. It scares me when I have trouble getting my breath.	0.64	0.42
11. When my stomach hurts, I worry that I might be really sick.	0.58	0.34
14. Unusual feelings in my body scare me.	0.68	0.46
18. Funny feelings in my body scare me.	0.58	0.33
*Factor 2. Cognitive concerns*		
2. When I cannot keep my mind on my schoolwork, I worry that I might be going crazy.	0.70	0.49
12. It scares me when I cannot keep my mind on my schoolwork.	0.66	0.44
15. When I am afraid, I worry that I might be crazy.	0.67	0.45
16. It scares me when I feel nervous.	0.61	0.38
*Factor 3. Social concerns*		
1. I do not want other people to know when I feel afraid.	0.39	0.15
5. It is important for me to stay in control of my feelings.	0.39	0.16
7. It embarrasses me when my stomach growls.	0.49	0.24
13. Other kids can tell when I feel shaky.	0.59	0.35
17. I do not like to let my feelings show.	0.28	0.08

Note. Factor names are shown in italics.

**Table 5:** Confirmatory factor analysis: goodness-of-fit indices for multigroup gender invariance of Model 1 (*N* = 1655).

	*S*-*Bχ*^2^/*gl*	GFI	CFI	SRMR	RMSEA	AIC
Equal forms (configural)	2.08	0.845	0.974	0.057	0.037 (0.032–0.041)	23.8
Equal factor loadings (measurement)	2.07	0.840	0.972	0.059	0.037 (0.033–0.041)	24.1
Equal factor variances and covariances (structural)	2.02	0.837	0.973	0.059	0.036 (0.032–0.040)	8.7

Note. Corrected indices (robust method). *S*-*Bχ*^2^—Satorra–Bentler scaled *χ*^2^; *gl*—degrees of freedom; GFI—goodness-of-fit index; CFI—comparative fit index; SRMR—standardized root mean square residual; RMSEA—root mean square error of approximation; and AIC—Akaike’s information criterion. The robust method was not used to compute the GFI and SRMR indices.

**Table 6:** Means and standard deviations (*SDs*) of the CASI as a function of sex.

	Total (*N* = 1655)	Boys (*n* = 800)	Girls (*n* = 855)	Boys vs. Girls
CASI Variable (Score Range)	Mean *(SD)*	Mean *(SD)*	Mean *(SD)*	*t* (1653)
Physical concerns (9–27)	14.7 (3.6)	13.7 (3.5)	15.6 (3.6)	10.3 ***
Cognitive concerns (4–12)	5.5 (1.6)	5.3 (1.5)	5.7 (1.7)	5.6 ***
Social concerns (5–15)	8.7 (1.9)	8.4 (1.9)	9.1 (1.8)	7.6 ***
CASI total (18–54)	28.9 (5.8)	27.4 (5.5)	30.3 (5.7)	10.7 ***

Note. *** *p* < 0.001.

## Data Availability

Participants only agreed to share their data with the researchers involved in this study. Therefore, datasets are not publicly available. If you would like to receive access, please contact B.S. (bsandin@psi.uned.es).

## Appendix A

**Table A1:** Percentiles (cut points) for scores on the CASI subscales and on the total scale.

	CASI Total			CASI Physical			CASI Cognitive			CASI Social		
Percentile	♂	♀	♂ + ♀	♂	♀	♂ + ♀	♂	♀	♂ + ♀	♂	♀	♂ + ♀
5	20	21	20	9	10	9	4	4	4	5	6	6
10	21	23	21	9	10	10	4	4	4	6	7	6
15	22	24	23	10	11	11	4	4	4	6	7	7
20	22	25	24	11	12	11	4	4	4	7	8	7
25	23	26	25	11	13	12	4	4	4	7	8	7
30	24	27	25	11	14	12	4	5	4	7	8	8
35	25	28	26	12	14	13	4	5	5	8	8	8
40	25	29	27	12	15	14	4	5	5	8	8	8
45	26	29	28	13	15	14	5	5	5	8	9	8
50	27	30	29	13	16	15	5	5	5	8	9	9
55	27	31	29	14	16	15	5	6	5	9	9	9
60	28	31	30	14	17	16	5	6	5	9	9	9
65	29	32	31	15	17	16	5	6	6	9	10	9
70	30	33	32	15	17	17	6	6	6	9	10	10
75	31	34	33	16	18	17	6	7	6	10	10	10
80	32	35	34	17	18	18	6	7	7	10	11	10
85	33	36	35	17	19	18	7	8	7	10	11	11
90	35	38	37	18	20	19	7	8	8	11	11	11
95	37	40	39	20	22	21	9	9	9	12	12	12

Note. Boys (♂) *n* = 800; girls (♀) *n* = 855; total sample (♂ + ♀) *N* = 1655. Score range: physical concerns, 9–27; cognitive concerns, 4–12; social concerns, 5–15; and CASI total, 18–54.

**Table A2:** Spanish version of the Childhood Anxiety Sensitivity Index (CASI) *.

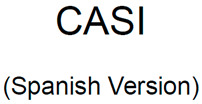

Instrucciones: A continuación se indican algunas frases que los chicos y chicas utilizan para describirse a sí mismos. Lee detenidamente cada frase y marca cada una de ellas en el cuadro correspondiente a una de las tres alternativas (Nada, Un poco, Mucho). Marca la que mejor se ajuste a tus sensaciones personales. No existen contestaciones buenas ni malas. Recuerda que únicamente tienes que señalar la palabra que mejor se ajuste a la manera en que te has sentido últimamente.			
Cuando tengo miedo, prefiero que la gente no se dé cuenta	☐ nada	☐ un poco	☐ mucho
2. Cuando no puedo concentrarme en mis deberes de clase, me preocupa que pueda estar volviéndome loco/a	☐ nada	☐ un poco	☐ mucho
3. Me asusto cuando siento que tiemblo	☐ nada	☐ un poco	☐ mucho
4. Me asusto cuando siento como si me fuera a desmayar	☐ nada	☐ un poco	☐ mucho
5. Para mí es importante poder controlar mis emociones	☐ nada	☐ un poco	☐ mucho
6. Me asusto cuando mi corazón late rápidamente	☐ nada	☐ un poco	☐ mucho
7. Me siento violento/a cuando mi estómago hace ruidos	☐ nada	☐ un poco	☐ mucho
8. Me asusto cuando siento como si fuera a vomitar	☐ nada	☐ un poco	☐ mucho
9. Cuando noto que mi corazón late rápido, me preocupa que pueda tener algo malo	☐ nada	☐ un poco	☐ mucho
10. Me asusto cuando no respiro bien	☐ nada	☐ un poco	☐ mucho
11. Cuando me duele el estómago, me preocupa que pueda estar realmente enfermo/a	☐ nada	☐ un poco	☐ mucho
12. Me asusto cuando no puedo concentrarme en los deberes de clase	☐ nada	☐ un poco	☐ mucho
13. Cuando noto que tiemblo, me preocupa que otros chicos/as también puedan darse cuenta	☐ nada	☐ un poco	☐ mucho
14. Me asusto cuando noto en mi cuerpo sensaciones nuevas o poco habituales	☐ nada	☐ un poco	☐ mucho
15. Cuando tengo miedo, me preocupa que pueda estar loco/a	☐ nada	☐ un poco	☐ mucho
16. Cuando estoy nervioso, me asusta no poder concentrarme en lo que estoy haciendo	☐ nada	☐ un poco	☐ mucho
17. Prefiero no mostrar mis sentimientos a los demás	☐ nada	☐ un poco	☐ mucho
18. Me asusto cuando siento en mi cuerpo sensaciones raras o inesperadas	☐ nada	☐ un poco	☐ mucho

Note. ***** Silverman et al., 1991 [15]. Adapted by B. Sandín, 1997, *Ansiedad, Miedos y Fobias en Niños y Adolescentes [Anxiety, Fears and Phobias in Children and Adolescents]*, p. 229, Dykinson Reproduced with permission.
